# Toolkits and Toolboxes on Cancer Care and Mental Health: A Scoping Review Following the PRSMA-ScR Guidelines

**DOI:** 10.3390/healthcare14142222

**Published:** 2026-07-22

**Authors:** Cristina Pena-Vargas, Laura Rivera-Ruiz, Fabiola N. Martinez-Pesante, Lianel P. Rosario, Paola del Río-Rodríguez, Guillermo Laporte-Estela, Jovanny Díaz-Rodríguez, Gabriela N. Marrero-Quiñones, Zindie Rodríguez-Castro, Cynthia Cortes-Castro, Ana Paula Cupertino, Normarie Torres-Blasco, Eliut Rivera-Segarra, Julio Jiménez, Rosario Costas-Muñiz, Guillermo N. Armaiz-Pena, Eida M. Castro-Figueroa

**Affiliations:** 1 School of Brain and Behavioral Sciences, Ponce Health Sciences University, Ponce, PR 00716, USA; lrosario21@stu.psm.edu (L.P.R.); pdelrio22@stu.psm.edu (P.d.R.-R.); glaporte22@stu.psm.edu (G.L.-E.); normarietorres@psm.edu (N.T.-B.); elrivera@psm.edu (E.R.-S.); jcjimenez@psm.edu (J.J.); ecastro@psm.edu (E.M.C.-F.); rcostas@psm.edu (R.C.-M.); 2 Ponce Research Institute, Ponce, PR 00716, USA; lrivera14@stu.psm.edu (L.R.-R.); fmartinez16@stu.psm.edu (F.N.M.-P.); jovadiaz@psm.edu (J.D.-R.); gmarrero22@stu.psm.edu (G.N.M.-Q.); zrodriguez@psm.edu (Z.R.-C.); ccortes@psm.edu (C.C.-C.); garmaiz@psm.edu (G.N.A.-P.); 3 Department of Surgery, University of Rochester, Rochester, NY 14642, USA; paula_cupertino@urmc.rochester.edu; 4 Department of Basic Sciences, Division of Pharmacology, School of Medicine, Ponce Health Sciences University, Ponce, PR 00716, USA; 5 School of Dental Medicine, Ponce Health Sciences University, Ponce, PR 00716, USA; 6 Department of Psychiatry, School of Medicine, Ponce Health Sciences University, Ponce, PR 00716, USA

**Keywords:** toolbox, toolkit, cancer care, mental health

## Abstract

**Background:** Toolkits/toolboxes represent a promising intervention tool for cancer care and mental health. However, research is needed to identify their key components and effective implementation strategies. **Methods:** Following PRISMA-ScR, the search was conducted across five databases using key terms. Included studies addressed mental health or cancer care using toolboxes or toolkits for adults, documented their delivery or implementation strategies, and were experimental or quasi-experimental in Spanish or English. Excluded studies involved adaptations, relied solely on qualitative methods, did not address the specified population, or were systematic reviews. Three independent reviewers conducted title/abstract screening, full-text review, and data charting, with discrepancies resolved by a senior reviewer. The review protocol was prospectively published in the Open Science Framework (OSF). The Downs and Black’s checklist was used to evaluate quality and assess for bias. **Results:** A total of 33 studies were included in the final review. The toolkits/toolboxes primarily consisted of themes related to mental health and wellness in cancer care. Most mental health diagnoses reported consisted of depression or depressive symptomatology (14, 20.3%) followed by anxiety (12, 17.4%). Educational (13, 15.1%) and cognitive and behavioral strategies (13, 15.1%) were the most frequently used, followed by self-management (10, 11.6%). A total of 13 (39.3%) included Hispanics/Latinos in their sample. In terms of quality appraisal, representation of the entire population was the least frequently met criterion (10, 30%). **Conclusions:** This review identified important methodological gaps in implementation and population representation, as well as intervention components that can represent promising key aspects of toolkits/toolboxes.

## 1. Introduction

Toolkits have been used to inform and improve health behaviors among various audiences, including healthcare professionals, patients, community and health organizations, policymakers, and the public. The term has been used to describe the bundling of a combination of educational materials, including templates, instruction sheets, literature reviews, videos, and posters, presented in various formats, such as hard copy or web pages [1]. As defined by the Agency for Healthcare Research and Quality (AHRQ), a toolkit is “an action-oriented compilation of related information, resources, or tools that together can guide users in developing a plan or organizing efforts to conform to evidence-based recommendations or meet specific evidence-based practice standards” [2]. Toolkits are designed to provide directions that guide users through the development of plans and organizing efforts to perform specific tasks, offering proactive recommendations and tools (e.g., surveys, guidelines, or checklists) [3]. Toolkits offer an innovative format to present an intervention with greater flexibility of use. They are a packaged group of tools and strategies that present explicit knowledge (e.g., templates, pocket card guidelines, algorithms) and are used to educate and/or facilitate behavior change [4]. The strategies in the toolkit/toolboxes are intended to be evidence-based, facilitate practice change, and may include methods for guideline implementation, informing policy, training practitioners, and providing quality audit materials [4]. The goal of the toolkit is for the user to select and use strategies at their discretion, depending on their aims, resources, and context. Toolkits have a popular appeal because they can be easily distributed on the Internet in an engaging and multimodal manner to a wide variety of audiences [1].

Given their practicality, toolkits could be a promising tool for managing chronic conditions such as cancer, depression, anxiety disorders, multiple sclerosis, dementia, bipolar disorder, and substance use disorders, among others. However, it is essential to examine which components drive effectiveness across different populations. This is especially relevant in behavioral health, as the rise in digital tools [5] has coincided with growing attention to intervention toolkits in the literature. Yet, a standardized toolkit format has not been established in the health context, underscoring the need for further evaluation and research interventions and implementation. It is also essential to consider diverse populations whose cultural and contextual factors influence how they respond to this type of intervention [5,6], as well as those who have not yet had access to such approaches. Likewise, beyond adapting intervention content, it is equally important to consider adaptation of the implementation process to ensure reach, engagement, and effectiveness [7].

The appropriateness of the intervention components and reach of toolkits/toolboxes may vary across populations due to differences in context. As such, there is a critical need to ensure that these interventions are inclusive of populations that have been underrepresented in research. For instance, in the cancer care context, cancer is the leading cause of death among Latinos in the United States, in contrast to the general U.S. population, for whom it ranks second [8,9]. Despite some cancers showing lower overall incidence rates among Hispanics/Latinos compared to non-Hispanic Whites, this population experiences disproportionately higher rates of infection-related cancers, including stomach, liver, and cervical cancer [10]. Latina women, for instance, face a notably higher incidence of cervical cancer compared to non-Hispanic White women, while Hispanic individuals as a whole experience higher rates of liver and gallbladder cancers, many of which are both preventable and linked to modifiable risk factors [10]. These disparities are compounded by lower rates of early detection, with data showing that Hispanic women are less likely to be diagnosed with breast cancer at a localized stage compared to non-Hispanic White women, reflecting gaps in cancer screening access and follow-through [9,10]. Moreover, the poor clinical outcomes observed in Hispanic/Latino populations are driven not only by biological or behavioral factors but also by deeply rooted structural inequities, including limited access to culturally tailored care, language barriers, lack of health insurance, and lower rates of enrollment in clinical trials, all of which compromise the continuum of cancer care [8,9]. In response to these challenges, toolkits/toolboxes are a promising strategy that may help incorporate educational interventions, streamlining the collection of resources, supporting informed decision-making, facilitating behavior change, and improving access to culturally relevant mental health and cancer care services. Hence, this scoping review’s primary aims are to (1) identify existing toolkits/toolboxes design to address mental health and cancer survivorship, (2) identify the intervention component incorporated in the toolkits/toolboxes, and (3) identify implementation factors of the toolkits/toolboxes, including strategies and delivery. Also, as secondary aims, this review seeks to (1) identify which toolboxes or toolkits include a Hispanic/Latino sample and how the toolkits are adapted to this population, and (2) evaluate the methodological quality of the studies. Findings from this review will inform the future development and adaptation of toolkit-based interventions.

## 2. Materials and Methods

### 2.1. Eligibility Criteria

The eligibility criteria established were developed to address the review’s aims. Eligibility was determined based on the characteristics of the intervention as reported by the study authors. Studies were included if they addressed mental health conditions as described by the authors or cancer survivorship issues. Studies had to report the use of a toolbox or toolkit. Studies were eligible if they documented implementation factors such as the delivery method (in person, web-based, app, etc.) and/or implementation strategies of the toolbox or toolkit, employed an experimental or quasi-experimental design, and were written in either Spanish or English. Studies that only described an adaptation of cancer survivorship or mental health toolboxes or toolkits, did not provide toolboxes to the population of interest, or were systematic review articles were excluded. Qualitative studies were excluded, as the purpose of this review was to identify and characterize interventions using toolkits or toolboxes and to examine how these interventions were delivered and implemented within experimental and quasi-experimental study designs, allowing for consistent evaluation of the characteristics of the implemented interventions across studies.

### 2.2. Information Sources and Search Strategy

This scoping review was conducted in accordance with the Preferred Reporting Items for Systematic Reviews and Meta-Analyses Extension for Scoping Reviews (PRISMA-ScR) [11] and using the Covidence platform from 14 November 2023 to 12 December 2023. A secondary search was conducted between 31 August and 4 September 2025 to update the review and capture articles published from 2024 to 2025 using the primary databases. The protocol for this scoping review was registered in the Open Science Framework (OSF). This review constituted a search of randomized controlled trials and quasi-experimental single-arm pre–post intervention trials using toolkits/toolboxes specifically addressing adults’ mental health and cancer patients and survivors. The research team first used the following primary databases: EBSCOhost Research Platform (EBSCOhost), PubMed, and Scientific Electronic Library Online (Scielo). Secondly, the team used Google Scholar and the Red de Revistas Científicas de América Latina y el Caribe, España y Portugal (Redalyc). In PubMed, the search strategy was conducted using MeSH (Medical Subject Headings), the National Library of Medicine’s controlled vocabulary thesaurus used for indexing articles in PubMed [12] and Boolean operators. Keyword-based searches using Boolean operators were employed in the remaining databases. The search included the key terms Hispanic, Latinx, Latino, cancer patients, cancer survivors, cancer caregivers, cancer care, toolkit, toolbox, mental health, and prevention. Lastly, Google Scholar was used, as it helps capture citations that may be missed by primary indexing sources. The search was limited to the first 200 results due to the large volume of records and the non-transparent ranking algorithm. This pragmatic cut-off is consistent with recommended practice for using Google Scholar in evidence synthesis, where screening is typically limited to a manageable number of results because lower-ranked records are less likely to yield additional relevant studies. We also applied basic search operators (e.g., inclusion and exclusion terms) to refine the results. All articles published up to September 2025 were included in this scoping review. To see the full search strategy, see Appendix A.

### 2.3. Selection of Sources of Evidence

We used Covidence (Veritas Health Innovation, Melbourne, Australia) software to manage all the screening procedures. All reviewers involved in the screening, data charting, and quality appraisal stages hold graduate-level training or established expertise in health sciences, psycho-oncology, behavioral health research, or scientific writing, providing the disciplinary grounding required for rigorous and contextually informed appraisal throughout the review. All titles and abstracts retrieved from the initial search were screened for relevance based on the established inclusion and exclusion criteria. Three independent reviewers (L.P.R., F.M.P. and L.R.R.) screened titles and abstracts based on predefined criteria, resolving discrepancies through discussion or consultation with a third reviewer when needed. Full-text articles were then assessed for eligibility using the same criteria.

### 2.4. Data Charting and Data Items

Data charting was performed using a standardized form developed a priori and implemented within Covidence, which allows for customized data extraction forms and facilitates independent extraction by multiple reviewers. We created a data charting template in Covidence, which included the study’s characteristics and the review’s outcomes. The data charting procedure followed the same eligibility process, where the data charting was resolved through discussion and consensus of three independent reviewers (L.P.R., F.M.P. and L.R.R.), and a third reviewer (C.P.V. and E.C.F.) resolved any discrepancies. The data items were bibliography, sample size, participants’ nationality/ethnicity, participants’ age, diagnosis, participant type, study design, inclusion criteria, intervention, delivery method, implementation strategy, and toolkit/toolbox intervention components. Although critical appraisal is not mandatory in scoping reviews, we included a methodological quality assessment to provide contextual information regarding the rigor of the identified intervention studies, proving an overview of the methodological strengths and limitations of the existing evidence. To do so, we used a modified version of Downs and Black’s checklist, which was shorten by the authors for feasibility [13]. This check list is a standardized tool for evaluating methodology quality for both randomized and non-randomized studies. Lastly, two reviewers (L.R.R. and F.M.P) independently conducted the critical appraisal for the quality of the studies, and any disagreement was resolved through discussion with a third reviewer (C.P.V. and E.C.F.).

### 2.5. Study Framework

The review is guided by the PIO framework (a variant of the PICO framework) [14], which focuses on Population, Intervention, and Outcomes. In this review, the Population includes cancer patients and survivors, cancer caregivers, and individuals with mental health conditions. The Intervention encompasses cancer survivorship and mental health toolkits or toolboxes. The Outcomes include implementation-related factors, intervention components, and overall study quality. In addition, the Expert Recommendations for Implementing Change (ERIC) framework was incorporated to guide the identification and categorization of implementation strategies relevant to this study [15].

### 2.6. Descriptive Analyses and Synthesis

Descriptive data (frequency, percentages, and central distribution statistics) analyses were conducted to evaluate all the study’s outcomes. The team utilized the IBM SPSS version 29 platform to conduct the analyses. Minimal missing data were identified. However, when the mean age of participants was not included, it was coded as missing. These studies were kept in the analysis, as this missing information was minimal and did not impact the main outcomes of the review. Researcher triangulation was conducted to evaluate content in the studies and determine categories regarding the implementation component. The intervention content was evaluated by three coders (J.D.R., G.M.Q., and C.P.V). The identified categories were discussed until consensus was reached. For the mapping of implementation strategies, a thematic analysis was conducted by a single coder using a predefined coding template based on the ERIC framework. Lastly, the team did not conduct a sensitivity analysis given the descriptive nature of the synthesis.

## 3. Results

### 3.1. Identification of Studies

**Figure 1:**
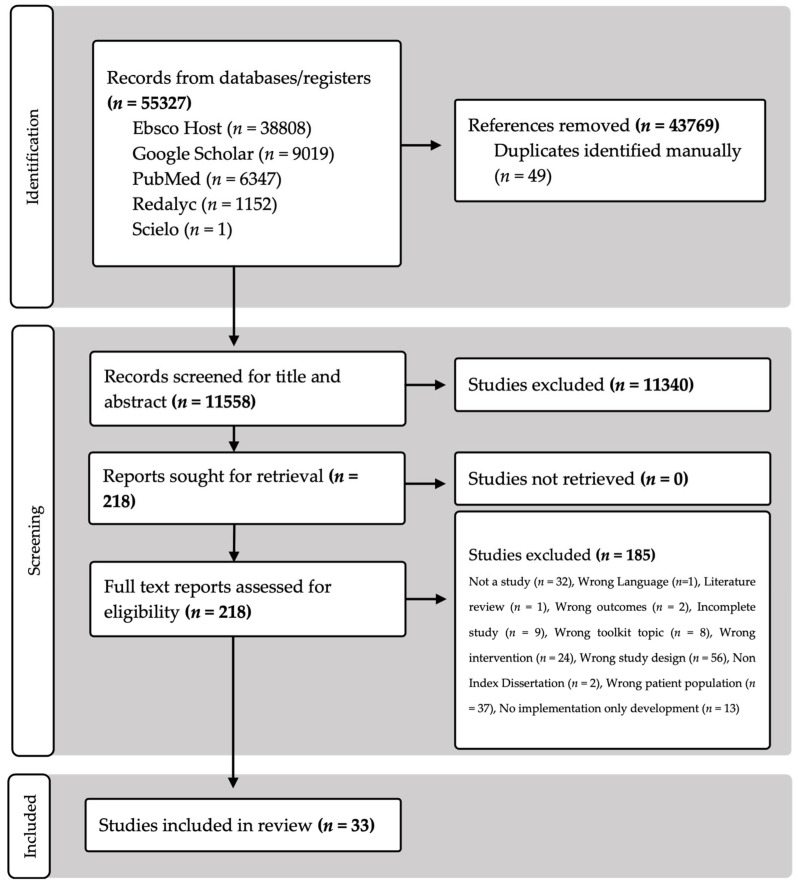
PRISMA flowchart.

A search was conducted across five databases, including EBSCOhost (*n* = 38,808), Google Scholar (*n* = 9019), PubMed (*n* = 6347), Redalyc (*n* = 1152, Spanish articles), and Scielo (*n* = 1, Spanish articles) (Figure 1). We searched five databases and found 55,327 records. After removing duplicates, 11,558 records remained and were screened based on their abstract. A total of 218 reports were selected for full review. Following a thorough screening process, we identified 33 studies for risk bias evaluation that focused on addressing the needs of adult cancer patients, mental health patients, and cancer survivors within the Hispanic population. These studies utilized toolboxes or toolkits tailored to specifically address mental health or cancer survivorship issues and document their respective delivery methods and implementation strategies.

### 3.2. General Description of the Studies

The mean age of participants across studies was $\overset{¯}{x}$ = 48.63 (SD = 24.42). The interventions (toolkits/toolboxes) addressed various themes, including mental health (15/33, 45.5%) [16,17,18,19,20,21,22,23,24,25,26,27,28,29,30], wellness in cancer care (12/33, 36.4%) [31,32,33,34,35,36,37,38,39,40,41,42], mental health in cancer care (2, 6.1%) [43,44], wellness and mental health (1/33, 3.0%) [45], substance use disorder (1/33, 3.0%) [46], wellness (1/33, 3.0%) [47], and symptom management in cancer care (1/33, 3.0%) [48].

### 3.3. Mental Health Diagnosis and Other Psychological Outcomes

Not all mental health- or cancer- and mental health-focused studies disclosed a diagnosis. Most of the samples consisted of depression or depressive symptomatology (14/33, 42.4%) [16,19,20,21,22,24,25,29,30,31,35,36,37,43], followed by anxiety (12/33, 36.3%) [16,19,22,24,30,31,36,37,42,43,44,45], severe mental illness (3/33, 9.0%) [17,18,27], substance abuse (2/33, 6.0%) [27,46], dementia (1/33, 3.0%) [28], autism (1/33, 3.0%) [26], and somatoform disorders (1/33, 3.0%) [27]. Other psychological and health outcomes that were not diagnosed comprised quality of life (12/33, 36.3%) [27,29,31,33,34,35,37,38,39,40,42,43], wellbeing (6/33, 18.1%) [16,22,36,38,40,47], distress (4/33, 12.1%) [23,36,40,42], fatigue (2/33, 6.0%) [35,42], and poor functioning (2/33, 6.0%) [24,35].

### 3.4. Intervention Components Incorporated and Implementation Factors

The team identified a total of 20 types of intervention components and resources used within the toolkits/toolboxes. Educational (13/33, 39.3%) [18,25,26,27,28,29,31,34,36,38,41,45,46] and cognitive and behavioral strategies (13/33, 39.3%) [16,17,19,20,21,22,25,27,29,30,38,44,45] were the most frequently used, followed by self-management (10, 11.6%) [18,22,25,27,31,32,39,41,43,48].

Other strategies included were psychoeducational (9/33, 27.2%) [16,17,22,23,24,30,39,43,44], support (6/33, 18.1%) [23,25,30,31,34,43], resources (5/33, 15.1%) [26,28,30,34,43], self-advocacy (4/33, 12.1%) [27,30,33,43], stress management (4/33, 12.1%) [28,30,36,42], symptom management (3/33, 9.0%) [35,43,48], mindfulness (3/33, 9.0%) [40,42,47], cognitive skills (3/33, 9.0%) [24,33,47], consultations (2/33, 6%) [32,37], interpersonal skills (2/33, 6.0%) [28,33], self-care (2/33, 6.0%) [28,39], psychosocial skills (2/33, 6%) [34,38], self-compassion (1/33, 3.0%) [22], coaching (1/33, 3.0%) [31], provider communication (1/33, 3.0%) [39], goal-setting (1/33, 3.0%) [31], and motivational enhancement (1/33, 3.0%) [46].

In terms of implementation strategies, considerable variability was observed. These are reported in the table due to their complexity and extent (See Table 1 for more information). We identified greater heterogeneity in contrast to the intervention components. Various delivery formats were used, including multiple modalities within the same study. Most papers did not provide a detailed description of their implementation approaches. Although some studies reported implementation processes, few clearly specified the strategies, frameworks, or theoretical basis guiding their approach. Only a small number of studies clearly outlined these elements in the manuscript [18,28] or referred to a previously published implementation of their intervention [23].

### 3.5. Inclusion of a Hispanic/Latino Sample and Adaptations

A total of 13/33 (39.3%) reported the inclusion of Hispanics/Latinos in their sample [17,24,26,29,32,34,36,38,40,41,42,44,48]. Nonetheless, out of the 13 studies that reported a Hispanic/Latino sample, a total of 8/33 (61.5%) reported a sample size of 10% to 1.7% [17,24,26,32,34,38,40,48]. Lastly, 4/33 (12.1%) [29,31,41,44] included implementation strategies that were aimed at tailoring their intervention to the Hispanic/Latino population.

In terms of tailoring intervention to the needs of Hispanics/Latinos, most interventions promoted adaptability [29,31,41,44] through the development or translation of intervention material into Spanish, including measurements in Spanish [29,31,41,44]. Additionally, some intervention materials or curricula were adapted to be culturally appropriate or to demonstrate cultural sensitivity [31,41]. One study supported adaptability using an advisory group composed of patients and providers who guided the development of the material [31]. Another intervention included an acculturation evaluation and reported the inclusion of bilingual staff [41]. Lastly, one study reported having facilitators in the community to support cultural competence [44].

### 3.6. Data Appraisal

Of the final 33 articles left for the risk bias evaluation, all 33 studies clearly described their hypothesis, aim, or objective and provided a clear description of their primary outcomes in either the introduction or methods section. A total of 32/33 (96.9%) [16,17,18,19,20,21,22,23,24,25,26,27,28,29,30,31,32,33,34,35,36,37,38,39,40,42,43,44,45,46,47,48] clearly described their main findings. A total of 30/33 (90.9%) included clearly described patient characteristics [16,17,18,19,20,21,22,23,24,25,26,27,28,29,30,31,32,33,34,35,36,37,38,39,40,41,42,43,44,48] and the intervention [16,17,18,19,20,21,22,23,24,25,26,27,28,29,30,31,33,34,35,36,37,38,39,40,41,42,43,44,46,47], and used the main outcome measures [16,17,18,19,20,21,22,23,24,25,27,28,29,30,31,32,33,34,36,37,38,39,40,41,42,43,44,45,46,47].

Regarding the distribution of the principal confounders, 20/33 (60.6%) [16,19,20,22,23,24,25,27,29,30,31,34,36,37,38,39,40,41,43,47] presented the distribution of each compared group clearly. Moreover, 18/33 (54.5%) [17,19,21,23,24,26,29,32,33,34,35,36,38,40,41,42,43,48] described the findings by race/ethnicity. However, 21/33 (63.6%) used randomization to allocate the subjects to the intervention groups [16,19,22,23,24,25,27,29,30,33,34,35,36,37,39,41,42,43,45,46,47]. Lastly, only a total of 10/33 (30.3%) [16,22,23,24,26,29,36,41,42,43] used subjects who were representative of the entire population from which they were recruited.

These findings show that most of the studies present adequate to moderate quality, as they clearly present and describe their objective, patient characteristics, intervention, and main findings. Nonetheless, just over half (54.5%) reported results stratified by race/ethnicity. Additionally, only 30.3% of studies included a sample that was representative of the populations from which the study was conducted, indicating potential concerns regarding external validity. These methodological observations suggest caution is warranted when interpreting their generalizability.

## 4. Discussion

Toolkits/toolboxes are developed to organize an intervention, deliver an intervention, and guide future dissemination and implementation efforts. The purpose of this scoping review was to examine the components of toolkits/toolboxes in the cancer care and mental context, including the resources, strategies, intervention approaches, and implementation processes incorporated. It also aimed to assess the extent to which these toolkits include Hispanic/Latino populations and account for their specific needs in the development or adaptation process. After the final screening phase of the 218 articles in the scoping review, only 33 studies remained, which can be viewed as a limited amount of literature available regarding the use of toolkits/toolboxes in mental health and cancer care contexts.

Mental health and wellness were the themes most incorporated into mental health and cancer care toolkits, underlying the relevance of integrating intervention components that fit these themes to better serve these populations. The team identified that the most addressed populations were patients with depression and anxiety, highlighting the value of using toolkits to address these mental health concerns. Although several intervention components and resources were observed within toolkits/toolboxes (e.g., self-management techniques, mindfulness, stress management, resources list), a consistent use of education and cognitive behavioral strategies was observed, each of which is strongly supported by existing literature. For instance, psychoeducation strategies for anxiety and depression have been seen to be beneficial for stress control [49,50,51,52]. Likewise, cognitive behavioral strategies have been proven to be beneficial for a variety of mental health diagnoses and concerns [53].

In contrast to the consistent use of components and themes, greater heterogeneity was observed in implementation factors, including the strategies employed and their delivery methods. Various delivery formats were used, even within the same studies, where a single toolkit/toolbox was often delivered through multiple modalities. However, it is important to consider how different delivery approaches may affect the target population and, accordingly, conduct prior needs assessments to identify the most appropriate delivery methods. In general, variability may compromise reproducibility and rigor when implementation processes are not clearly reported. In some cases, heterogeneity is not only advantageous but essential, as it is anticipated that interventions will need to be adapted and tailored to address the specific needs of diverse populations. Our findings show that most papers lacked an explicit rationale for their implementation strategies or a detailed implementation plan and omitted important implementation factors such as strategies, frameworks, or theoretical bases guiding their approach. As a result, implementation was often reported narratively rather than systematically. The lack of documentation represents a critical gap in the literature, particularly regarding toolkit standardization. Future efforts should focus on developing standardized protocols for toolkit development that retain flexibility and adaptability, while ensuring that implementation decisions are planned, justified, and clearly documented.

Regarding the applicability of toolkits across cancer types and stages, the evidence reviewed suggests that toolkit-based interventions hold potential across the full cancer care continuum. Patients may experience significant psychological distress at any point from initial diagnosis and active treatment through post-treatment survivorship and palliative care, underscoring the relevance of flexible, accessible interventions at each of these phases [54]. The studies included in this review spanned a range of cancer diagnoses, including breast, lung, colorectal, gynecologic, hematologic, head and neck, and prostate cancers, reflecting the cross-cutting applicability of toolkit approaches regardless of tumor type. Self-management interventions, a core component of the toolkits identified in this review, have been shown to be feasible not only during survivorship but also during the active treatment phase, where building patients’ self-management capacity may buffer against the psychological and functional burden of treatment [43]. Furthermore, at the palliative and end-of-life stages, toolkits that address pain management, caregiver support, and psychosocial coping have demonstrated feasibility and acceptability, as illustrated by studies included in this review. The modular format of toolkits, allowing users to select components according to their stage-specific needs, is particularly well suited to this continuum-based approach. Future work should address how toolkit components may need to be adapted not only across cultural groups but also across disease stages, given that the priorities, burdens, and resources of patients shift substantially along the cancer trajectory.

Moreover, a common limitation in many of these studies was the lack of inclusion of subjects that were representative of the entire population, which can compromise the generalizability of the findings and contribute to disparities, particularly for minority groups [55]. Although many studies reported nationality, some failed to include ethnicity breakdowns or described their findings based on race and/or ethnicity. Additionally, we observed a lack of inclusion of Hispanic/Latino individuals in the cohorts, as only 13 studies included this population, and only 5 of them reported a sample in which Hispanic/Latino participants made up more than 10%. This aligns with data from the US Food and Drug Administration, which shows that Hispanics account for 10% of participants in clinical trials for new drugs, and just 4.6% in trials for novel cancer therapies [56]. Barriers such as mistrust of the medical system, language challenges, limited access to academic cancer centers, and logistical issues (e.g., transportation, childcare, and work obligations) contribute to this underrepresentation [57]. Furthermore, the failure to report findings by race and ethnicity, omitted in approximately 45% of studies, further limits the ability to interpret results in a way that addresses disparities [58,59]. Addressing these methodological shortcomings is essential to ensure inclusive, empirically grounded science that can equitably serve all populations.

In light of these considerations, this review provides valuable insights into the use of toolkits/toolboxes for supporting individuals in managing mental health diagnoses and other health outcomes, such as quality of life, wellbeing, distress, and functionality problems. Furthermore, by identifying challenges and potential areas for future development, this work serves as a foundation for improving mental health and cancer care. Identifying methodological issues also contributes to a deeper understanding and development of comprehensive and appropriate toolkits/toolboxes for mental health and cancer patients. Lastly, this scoping review identifies key gaps and opportunities in the application of toolkits within cancer care, particularly those that incorporate behavioral and psychological components. The findings of this scoping review provide a foundation for future research aimed at systematically evaluating the effectiveness of patient-directed toolkits/toolboxes through rigorous systematic reviews and meta-analyses.

## 5. Strengths and Limitations

Several methodological strengths of this review merit consideration. The search strategy was systematic and pre-registered in the OSF, ensuring transparency and reducing reporting bias. The inclusion of five databases, among them two Spanish-language repositories (Redalyc and Scielo), reflects a deliberate effort to capture literature relevant to Hispanic/Latino populations that may not be indexed on English-language platforms alone. The bilingual search design, encompassing both English and Spanish terms, further strengthened the comprehensiveness of the search. The use of three independent reviewers at each stage of the screening, data charting, and quality appraisal process, with structured consensus procedures for resolving discrepancies, supported methodological rigor. The Downs and Black checklist provided a standardized approach to quality appraisal applicable to both randomized and non-randomized designs, which was appropriate given the heterogeneity of the study designs included. A secondary search conducted through 2025 ensured that recently published literature was captured within the review.

However, a limitation of this review is that the search strategy did not include some recommended databases for systematic reviews and scoping review (e.g., EMBASE, CENTRAL, Scopus). We prioritized PubMed and other databases that index psychological and behavioral interventions and studies involving Hispanic/Latino populations (Redalyc and Scielo), consistent with the focus of this review. While this approach ensured alignment with our research objectives, the exclusion of those databases may have reduced the comprehensiveness of the search and introduced the possibility that relevant studies were not identified. Another limitation of this review is that implementation strategies were identified and classified by a single reviewer using the ERIC framework, which may have introduced classification bias despite the use of standardized definitions.

## 6. Practical Implications

The findings of this review carry several practical implications for the development, implementation, and dissemination of toolkit-based interventions in cancer care and mental health. First, the evidence supports the development of standardized toolkit protocols that clearly document the rationale for implementation strategies, the theoretical frameworks guiding intervention delivery, and the criteria for component selection. Standardization does not preclude adaptability; rather, it provides a structured foundation from which practitioners can make informed and justified decisions about tailoring interventions to their specific populations and contexts. Toolkits that explicitly document their flexibility parameters alongside their core elements are more reproducible, more evaluable, and more easily disseminated across clinical and community settings.

Second, the low representation of Hispanic/Latino individuals across the identified studies, and the even lower proportion of toolkits that meaningfully incorporated culturally and linguistically appropriate adaptations, highlights a pressing need for the intentional development of toolkit interventions that are designed with and for this population from the outset, rather than adapted as an afterthought. This includes translation into Spanish, engagement of community stakeholders in the development process, incorporation of culturally relevant content, and attention to structural barriers to access such as transportation, literacy, and insurance status.

Third, the findings underscore the value of embedding toolkits within existing cancer care pathways rather than positioning them as standalone add-ons. Integration into survivorship care plans, primary care models, and community health worker programs may improve reach and sustainability. Finally, future research should prioritize reporting by race and ethnicity, ensure adequate representation of minority populations in study samples, and adopt clear implementation frameworks to enable meaningful cross-study comparisons and the eventual development of evidence-based guidelines for toolkit use in oncology settings.

## 7. Conclusions

This review highlights how toolkits/toolboxes can serve as a key tool for interventions in the cancer care context and the mental health field. It identified key challenges, especially those related to implementation, as well as methodological issues and gaps. In particular, we identified that most of the methodological problems within the toolkit/toolbox studies have implications for underrepresented groups and may contribute to health disparities among them. In particular, the criterion that proved to be most neglected was the underrepresentation of other racial and ethnic minority groups. However, this review identified consistent intervention components and resources that represent promising aspects within the toolkits/toolboxes, including key themes, outcome measures, and evidence-based strategies.

## Figures and Tables

**Table 1:** Description of studies selected.

Bibliography	Sample Size	Patient Nationality/Ethnicity	Participant Type	Study Design	Inclusion Criteria	Delivery Method	Implementation Strategies
[43] McCusker J, Jones JM, Li M, et al. CanDirect: Effectiveness of a Telephone-Supported Depression Self-Care Intervention for Cancer Survivors. J Clin Oncol. 2021;39(10):1150–1161. doi:10.1200/JCO.20.01802	*N* = 245	Canadian	Patients in cancer remission; breast, hematologic and lymphatic, genitourinary, gynecologic, other	Randomized controlled trial	Eligible participants were age 18 years or older, had completed primary treatment (surgery, radiation, and/or chemotherapy) for any cancer (adjuvant therapies permitted), had been diagnosed 1–10 years previously, were in remission, and had a Patient Health Questionnaire-9 depression score of 8–19, indicating mild to moderate depressive symptoms.	Paper-based and electronic toolkit, telephone-based guidance	Distribute educational material, facilitation
[41] Santiago-Torres, M.; Contento, I.; Koch, P.; Tsai, W.Y.; Brickman, A. M.; Gaffney, A. O.; Thomson, C. A.; Crane, T. E.; Dominguez, N.; Sepulveda, J.; et al. ¡Mi Vida Saludable! A Randomized, Controlled, 2 × 2 Factorial Trial of a Diet and Physical Activity Intervention among Latina Breast Cancer Survivors: Study Design and Methods. *Contemporary clinical trials* **2021**, 110, doi:10.1016/j.cct.2021.106524.	*n* = 167	Argentinian, Colombian, Cuban, Dominican, Ecuadorian, Salvadorian, Honduran, Mexican, Nicaraguan, Peruvian, Puerto Rican	Cancer-free subjects; breast cancer survivors	Randomized controlled trial	Female; 18 years old or older; self-identification as Latina or Hispanic; self-reported history of stage 0–III breast cancer; no evidence of recurrent or metastatic disease; at least 90 days post final chemotherapy, biologic therapy, or radiation treatment and/or breast surgery (current use of endocrine therapy permitted); nonsmoker (within the past 30 days); intake of <5 daily servings of fruits and vegetables and/or engaging in <150 min per week of MVPA, as assessed by brief screening questionnaires; willingness and ability to receive text messages via cell phone/smart phone and email newsletters via computer, tablet, or smartphone; and willingness and ability to attend four 4 h in-person group sessions and to travel to complete study activities and clinic visits at baseline, 6- and 12-month timepoints	In-person group and digital-based communication	Promote adaptability, develop educational material, conduct educational meetings, facilitate, involve patients/consumers and family members
[45] Soares, L.; Silva, C.; Lucas, C. V., Toolbox: Technology Anxiety Resource for University Students. *Preprints* **2023**, doi:10.20944/preprints202306.0452.v1.	*n* = 31	Portuguese	Mental health patients	Other: The methodology is experimental analysis pre- and post-intervention. The aim is to compare the pre- and post-intervention, where anxiety management exercises were implemented via an online platform (intervention group) with a group that was not the target of the intervention via an online plat- form (control group).	Not disclosed	Web-based	Develop educational materials, distribute educational material
[20] Fogarty, A. S.; Proudfoot, J; Whittle, E. L.; Clarke, J.; Player, M. J; Christensen, H.; Wilhelm, K. Preliminary Evaluation of a Brief Web and Mobile Phone Intervention for Men with Depression: Men’s Positive Coping Strategies and Associated Depression, Resilience, and Work and Social Functioning. *JMIR mental health* **2017**, *4*, doi:10.2196/mental.7769.	*n* = 144	Australian	Mental health patients	Other: NRCT; single-group repeated-measures design	Score of at least 5 or more on the Patient Health Questionnaire-9 (PHQ-9; i.e., mild depression), had a valid email address, could access the Internet via both a computer and a mobile phone, were age 18 years or older, were residents of Australia, and were comfortable reading and writing in English	Web-based	Distribute educational materials
[19] Fitzgeraldson, E.; Triandafilidis, Z.; Franklin, Y.; Palazzi, K.; Kay-Lambkin, F.; Fitzpatrick, S. Feasibility and Acceptability of a Novel Online Program for Mental Health Carers. *International journal of psychological research* **2023**, *16*, 41–55, doi:10.21500/20112084.5733.	*n* = 108	Australian (Indigenous Australian, Aboriginal, Torres Strait Islander); other country of birth: United Kingdom, Egypt, Germany, India, New Zealand, South Africa, Zambia	Caregivers of mental health patients	Other: pilot RCT; two-arm randomized controlled trial	Age 16 years or older, supporting a person (of any age) experiencing depressive or anxiety symptoms that were impacting their life (formal diagnosis not required), living in Australia, comfortable reading and writing in English and using web-based programs	Web-based	Distribute educational materials, obtain and use feedback from patients/consumers and family
[44] Sánchez-Sosa, J. J.; Alvarado Aguilar, S. A Behavioral Self-Recording Procedure in the Management of Breast Cancer: A Field Test with Disadvantaged Participants. *Revista mexicana de análisis de la conducta* **2008**, *34,* 313–331.	*n* = 6	Mexican	Patients with breast cancer	Other: pre–post-test measures	Confirmed diagnosis of breast cancer with at least three months of evolution and recipients of healthcare services at the main hospital of Mexico’s National Cancer Institute	Paper-based handouts	Promote adaptability, provide clinical supervision, distribute educational materials, provide ongoing consultation, model and simulate change
[17] Buck, B.; Nguyen, J.; Porter, S.; Ben-Zeev, D.; Reger, G. M. FOCUS mHealth Intervention for Veterans With Serious Mental Illness in an Outpatient Department of Veterans Affairs Setting: Feasibility, Acceptability, and Usability Study. *JMIR mental health*, **2022**, *9*, doi:10.2196/26049.	*n* = 17	North American; race: American Indian or Alaskan Native, Asian, Black or African American, White; ethnicity: Hispanic or Non-Hispanic	Mental health patients	Other: NRCT; pilot feasibility study	Had a serious and chronic mental illness (e.g., schizophrenia spectrum or mood disorder) with current or past psychotic symptoms and received services at the PRRC	Mobile app	Distribute educational materials, obtain and use feedback from patients/consumers and family
[32] Cairo, J.; Williams, L.; Bray, L.; Goetzke, K.; Perez, A. C. Evaluation of a Mobile Health Intervention to Improve Wellness Outcomes for Breast Cancer Survivors. *Journal of patient-centered research and reviews* **2020**, *7*, 313, doi:10.17294/2330-0698.1733.	*n* = 127	North American; race: Asian, Black/African American, White; ethnicity: Hispanic and Non-Hispanic	Patients with breast cancer	Other: non-randomized experimental study; 2 group control study design	Women 18 years of age or older with curative-intent (stage 0–III) breast cancer who were not currently enrolled in any other wellness studies	Mobile app and paper-based handouts	Facilitation, distribute educational materials
[35] Hodge, F. S.; Line-Itty, T.; Arbing, R. H. Cancer-Related Symptom Management Intervention for Southwest American Indians. *Cancers* **2022**, *14*, 4771, doi:10.3390/cancers14194771.	*n* = 222	American Indians	Patients with cancer; patients in cancer remission; mental health patients; males: prostate, colon/rectal, stomach and lung cancer; females: breast cancer, ovarian, colorectal and kidney cancer	Randomized controlled trial	Being American Indian, age 18 or older, diagnosed with cancer by a medical provider, and residents of the state of Arizona	In-person group and paper-based handouts	Facilitation, distribute educational materials
[48] Spoelstra, S. L.; Sikorskii, A.; Majumder, A.; Burhenn, P. S.; Schueller, M.; Given, B. Oral Anticancer Agents: An Intervention to Promote Medication Adherence and Symptom Management. *Clinical journal of oncology nursing* **2017**, *21*, 157–160., doi:10.1188/17.CJON.157-160.	*n* = 54	North American	Patients with cancer; specific cancer type not disclosed	Other: Phase 1 refined the ADHERE intervention using an iterative single-subject design, which has previously proven effective in practice-based research. The intervention was used with one patient and improved prior to use with the next patient. Phase 2 determined feasibility, preliminary efficacy on adherence, symptom severity, and satisfaction using a quasi-experimental, longitudinal, sequential design over eight weeks. To prevent control group contamination, patients were enrolled in the control group first, followed by the intervention group.	Age 21 years or older, prescribed an OA within the past 30 days, and able to speak and read English	In-person individual and telephone-based guidance	Conduct educational meetings, distribute educational material
[38] Nahm, E. S.; Miller, K.; McQuaige, M.; Corbitt, N.; Jaidar, N.; Rosenblatt, P.; Zhu, S.; Son, H.; Hertsenberg, L.; Wickersham, K. E.; et al. Testing the Impact of a Cancer Survivorship Patient Engagement Toolkit on Selected Health Outcomes. *Oncology nursing forum* **2019**, *46*, 572–584, doi:10.1188/19.ONF.572-584.	*n* = 30	North American; race: Black, White, other ethnicity: Non-Hispanic or -Latino, Hispanic or Latino	Cancer-free subjects; breast cancer survivors	Other: NRCT; one-group pre–post design	Age 18 years or older, diagnosed with cancer, and treated with curative intent within six months from enrollment, able to use the Internet and email independently, had access to the Internet and email, and either had an existing patient portal account or signed up for one prior to the start of the study	Web-based	Distribute educational materials, facilitation
[34] Duggleby, W.; Ghosh, S.; Struthers-Montford, K.; Nekolaichuk, C.; Cumming, C.; Thomas, R.; Tonkin, K.; Swindle, J Feasibility Study of an Online Intervention to Support Male Spouses of Women With Breast Cancer. *Oncology nursing forum* **2017**, *44*, 765–775, doi:10.1188/17.ONF.765-775.	*n* = 57	Canadian; Caucasian, First Nations, Middle Eastern, Asian, and Chilean	Patients with breast cancer; caregivers of patients with cancer	Randomized controlled trial	Patient’s partner: age 18 years or older, being male, English-speaking, and currently living with and in a legal relationship with a woman (defined as married or cohabiting for a minimum of one year) with breast cancer (stage I–III); breast cancer patient: age 18 years or older, female, English-speaking, and diagnosed with breast cancer (stage I–III)	Web-based	Distribute educational materials, obtain and use feedback from patients/consumers and family
[42] Stoerkel, E.; Bellanti, D.; Paat, C.; Peacock, K.; Aden, J.; Setlik, R.; Walter, J.; Inman, A. Effectiveness of a Self-Care Toolkit for Surgical Breast Cancer Patients in a Military Treatment Facility. *The Journal of Alternative and Complementary Medicine* **2018**, *24*, 916–925, doi:10.1089/acm.2018.0069.	*n* = 100	North American	Patients with breast cancer; mental health patients	Randomized controlled trial	Women older than 18 years who were newly diagnosed with nonmetastatic breast cancer and for whom surgery (e.g., lumpectomy or mastectomy) would be their initial treatment	Paper-based handout, audio files and additional tools (wristband and journal)	Conduct educational meetings, distribute educational materials, obtain and use feedback from patients/consumers and family
[28] Smith, C. W.; Graves, B.A. Implementation and Evaluation of a Self-Care Toolkit for Caregivers of Families with Dementia. *Journal of the American Association of Nurse Practitioners* **2020**, *33*, 831–837, doi:10.1097/JXX.0000000000000469.	*n* = 35	North American	Caregivers of mental health patients	Other: quasi-experimental study, with a pre- and post-survey/assessment before and after the intervention was implemented	Male and female spouses, in-laws, and adult children who were caregivers of a family member with dementia; provided direct care for patients with dementia age 50 years or older who also had at least one or more comorbidities, such as diabetes, hypertension, coronary artery disease, or chronic obstructive pulmonary disease; were 30 years of age or older; spoke English; lived with the patient with dementia; and were the primary caregiver	In-person Group	Conduct educational meetings, distribute educational materials, assess for readiness and identify barriers, audit and provide feedback
[18] Enrique, A.; Duffy, D.; Lawler, K.; Richards, D.; Jones, S. An Internet-Delivered Self-Management Programme for Bipolar Disorder in Mental Health Services in Ireland: Results and Learnings from a Feasibility Trial. *Clinical psychology & psychotherapy* **2020**, *27*, 925–939, doi:10.1002/cpp.2480.	*n* = 15	Irish	Mental health patients	Other: NRCT; an uncontrolled, within-group, pre–post design with embedded mixed-methods evaluation	Patient: age 18 years or older with access to a smartphone or computer and the internet, capacity to provide consent to participate, clinical diagnosis of BD Type I or II established by a psychiatrist. Clinician: belonging to the clinical team in charge of patients with BD, specialists in the care team (i.e., psychiatrist, doctor, nurse, social worker, psychologist, or occupational therapist).	Web-based	Conduct educational meetings, identify and prepare champions, distribute educational materials, remind clinicians, obtain and use feedback from patients/consumers and family Non-ERIC: user reminder
[33] Davis, C.; Rust, C.; Choi, S. A Pilot Randomized Study of Skills Training for African American Cancer Survivors. *Social work in public health* **2014**, *29,* 549–560, doi:10.1080/19371918.2014.892865.	*n* = 71	African-American/Black women	Patients with breast, lung, or rib cancer; cancer-free “survivor” subjects	Other: RCT pilot; randomized repeated-measures experimental design	Female, African American, self-identified as being diagnosed with breast cancer, within one year of acute treatment, at least 18 years of age, and provided written consent to participate	In-person group	Conduct educational meetings, facilitation
[27] O’Keeffe, D.; Hickey, D.; Lane, A.; McCormack, M.; Lawlor, E.; Kinsella, A.; Donoghue, O.; Clarke, M. Mental Illness Self-Management: A Randomised Controlled Trial of the Wellness Recovery Action Planning Intervention for Inpatients and Outpatients with Psychiatric Illness. *Irish journal of psychological medicine* **2016**, *33*, 81–92, doi.org/10.1017/ipm.2015.18.	*n* = 36	Not specified; Irish	Mental health patients	Randomized controlled trial	People age 18–65 with a diagnosis of a mental or behavioral disorder who were inpatients of the private hospital or outpatients of its outpatient service	In-person group	Facilitation, conduct educational meetings, tailor strategies
[26] Nicolaidis, C.; Raymaker, D.; McDonald, K.; Kapp, S.; Weiner, M.; Ashkenazy, E.; Gerrity, M.; Kripke, C.; Platt, L.; Baggs, A. The Development and Evaluation of an Online Healthcare Toolkit for Autistic Adults and Their Primary Care Providers. *Journal of general internal medicine* **2016**, *31*, 1180–1189, doi:10.1007/s11606-016-3763-6.	*n* = 259	North American	Mental health patients	Other: NRCT; mixed-method single-arm pre-/post-intervention comparison	Patient (cognitive interview): must be 18 years old or older, reside in the United States, and communicate in English. Additional eligibility criteria for autistic adults were a medical diagnosis on the autism spectrum (autistic disorder, Asperger’s, pervasive developmental disorder not otherwise specified, or autism spectrum disorder). Patient (toolkit study): have a designated PCP. PCP (cognitive interview): current primary care practice with adult patients. PCP (toolkit study): their patient participated in the study.	Web-based	Distribute educational materials, conduct local needs assessment, obtain and use feedback from patients/consumers and family
[46] Brooks, A. C.; Chambers, J. E.; Lauby, J.; Byrne, E.; Carpenedo, C. M.; Benishek, L. A; Medvin, R.; Metzger, D. S.; Kirby, K. C. Implementation of a Brief Treatment Counseling Toolkit in Federally Qualified Healthcare Centers: Patient and Clinician Utilization and Satisfaction. *Journal of substance abuse treatment* **2016**, *60*, 70–80, doi:10.1016/j.jsat.2015.08.005.	*n* = 600	Not disclosed	Mental health patients	Other: RCT pilot; hybrid effectiveness- implementation study	Scoring 8 or more on the Alcohol Use Disorder Identification Test (AUDIT) or scoring 2 or more on the Drug Abuse Screening Test (DAST)	In-person individual and paper-based handouts	Tailor strategies, distribute educational materials, identify and prepare champions, provide ongoing consultation, assess for readiness and identify barriers and facilitators, conduct educational meetings
[24] Lorenzo-Luaces, L.; Howard, J. Efficacy of an Unguided, Digital Single-Session Intervention for Internalizing Symptoms in Web-Based Workers: Randomized Controlled Trial. *Journal of medical Internet research* **2023**, *25*, doi:10.2196/45411.	*n* = 828	North American	Mental health patients	Other: randomized controlled trial; pure RCT	An affirmative answer (i.e., responding “yes”) to the question “Do you have—or have you had—a diagnosed, ongoing mental health/illness/condition?”	Web-based	Distribute educational materials
[47] Pogrebtsova, E.; Son Hing, L.; González-Morales, M. G. Effectiveness of an Emotion Regulation Intervention versus an Active Control on Daily Well-Being and Cognitive Reappraisal: An Experience Sampling Randomized Controlled Trial. *International Journal of Stress Management* **2022**, *29*, 400–412, doi:10.1037/str0000259.	*n* = 130	Not specified; Canadian	Mental health patients	Randomized controlled trial	Not disclosed	In-person group and web-based	Conduct educational meetings, distribute educational materials Non-ERIC: reminder for participants
[21] Kratz, A. L.; Alschuler, K. N.; Williams, D. A.; Ehde, D. M. Development and Pilot Testing of a Web-Based Symptom Management Program for Multiple Sclerosis: My MS Toolkit. *Rehabilitation psychology* **2021**, *66*, 224–232, doi:10.1037/rep0000375.	*n* = 20	Not specified; North American	Mental health patients	Other: pilot single-arm-trial. “The first study phase involved the development of a web-based symptom self-management program. The second phase involved a single-arm pilot trial.”	18 years or older; self-report a diagnosis of MS (all MS subtypes included, regardless of disease duration, disability level, or disease-modifying-therapy status), have access to a reliable Internet-connected device (e.g., computer, tablet), and read, speak, and understand English. Required to have at least one of the following: moderate/moderately severe depressive symptoms as indicated by a score of 4 on the Patient Health Questionnaire–2, chronic pain, as indicated by an average pain intensity of 3/10 over the past 3 months on the 0 –10 numerical rating scale and/or significant fatigue, defined as a score of 10 on the five-item Modified Fatigue Impact Scale–5.	Web-based	Distribute educational material, assess for readiness and identify barriers and facilitators Non-ERIC: reminder for participants
[29] Wells, K. B.; Jones, L.; Chung, B.; Dixon, E. L.; Tang, L.; Gilmore, J.; Sherbourne, C.; Ngo, V. K.; Ong, M. K.; Stockdale, S.; et al. Community-Partnered Cluster-Randomized Comparative Effectiveness Trial of Community Engagement and Planning or Resources for Services to Address Depression Disparities. *Journal of general internal medicine* **2013**, *28*, 1268–1278, doi:10.1007/s11606-013-2484-3	*n* = 1246	North American	Mental health patients	Randomized controlled trial	Limited to clients providing contact information and having at least mild depression (score ≥ 10 on the 8-item Patient Health Questionnaire (PHQ-8), with the same scoring characteristics and cut-point as the PHQ-9	Web-based, flash drive, paper-based handouts, and video files	Involve patients/consumers and family members, promote adaptability, tailor strategies, distribute educational materials, conduct educational meetings, provide ongoing consultation, develop and implement tools for quality monitoring, use train-the-trainer strategies
[23] Lobban, F.; Akers, N.; Appelbe, D.; Chapman, L.; Collinge, L.; Dodd, S.; Flowers, S.; Hollingsworth, B.; Johnson, S.; Jones, S.H.; et al. Clinical Effectiveness of a Web-Based Peer-Supported Self-Management Intervention for Relatives of People with Psychosis or Bipolar (REACT): Online, Observer-Blind, Randomised Controlled Superiority Trial. *BMC Psychiatry* **2020**, *20,* 160, doi:10.1186/s12888-020-02545-9.	*n* = 800	British	Caregivers of mental health patients	Other: randomized controlled trial; pure RCT	Age 16 or over, living in the UK, relative or close friend of someone with psychosis or bipolar, currently experiencing distress (selecting “rather more than usual” or “much more than usual” on GHQ-28 item “Have you recently been feeling nervous and strung up all the time?”), currently seeking help (self-identified), Internet access, sufficient English fluency to comprehend intervention content	Web-based	Distribute educational materials, facilitation, tailor strategies, promote adaptability, conduct educational meetings
[40] Salsman, J. M.; McLouth, L. E.; Tooze, J. A.; Little-Greene, D.; Cohn, M.; Kehoe, M. S.; Moskowitz, J.T. An eHealth, Positive Emotion Skills Intervention for Enhancing Psychological Well-Being in Young Adult Cancer Survivors: Results from a Multi-Site, Pilot Feasibility Trial. *International journal of behavioral medicine* **2023**, *30*, 639–650, doi:10.1007/s12529-023-10162-5.	*n* = 33	North American	Cancer-free subjects; brain, breast, colorectal, head and neck, Hodgkin lymphoma, leukemia, melanoma, non-Hodgkin lymphoma, ovarian, testicular, thyroid	Other: single-arm, multi-phase pilot feasibility trial of EMPOWER with two waves of data collection	Be able to read and understand English, ability to provide informed consent, have a past history of a cancer diagnosis (excluding basal cell skin carcinoma), be 18 to 39 years of age at diagnosis and currently be within 0–5 years post-active treatment, and have reliable internet access	Web-based	Obtain and use feedback from patients/consumers and family, distribute educational content Non-ERIC: reminder for participants
[25] McCusker, J.; Cole, M. G.; Yaffe, M.; Strumpf, E.; Sewitch, M.; Sussman, T.; Ciampi, A.; Lavoie, K.; Platt, R. W.; Belzile, E. A Randomized Trial of a Depression Self-Care Toolkit with or without Lay Telephone Coaching for Primary Care Patients with Chronic Physical Conditions. *General hospital psychiatry* **2015**, *37*, 257–265, doi:10.1016/j.genhosppsych.2015.03.007.	*n* = 223	Canadian	Mental health patients	Randomized controlled trial	Age 40 or older; at least one self-reported doctor-diagnosed chronic physical condition or chronic pain of at least 6 months’ duration, determined by single questions, and a positivePHQ-2 screen-a PHQ-9 score of 5 or more; those with mild depression symptoms (PHQ-9 5–9) were included because they are at increased risk of developing more severe symptoms; and ability to read in either English or French	Paper-based handouts, audio and video files, and telephone-based guidance	Distribute educational materials, facilitation, provide clinical supervision, audit and provide feedback, conduct educational meetings
[16] Bakker, D.; Kazantzis, N.; Rickwood, D.; Rickard, N. A Randomized Controlled Trial of Three Smartphone Apps for Enhancing Public Mental Health. *Behaviour research and therapy* **2018**, *109*, 75–83, doi:10.1016/j.brat.2018.08.003.	*n* = 226	Australian	Mental health patients	Randomized controlled trial	Not disclosed	Mobile app	Distribute educational materials, alter patient/consumer fees
[37] Mitchell, G. K.; Girgis, A.; Jiwa, M.; Sibbritt, D.; Burridge, L. H.; Senior, H. E. Providing General Practice Needs-Based Care for Carers of People with Advanced Cancer: A Randomised Controlled Trial. *The British journal of general practice: the journal of the Royal College of General Practitioners* **2013**, *63*, e683–e690, doi:10.3399/bjgp13X673694.	*n* = 392	Australian	Caregivers of patients with patients with locally invasive or metastatic disease; mental health patients	Randomized controlled trial	Participants were adult (≥18 years) carers of patients with locally invasive or metastatic disease, who were capable of providing informed consent.	In-person individual, digital-based handouts and paper-based handouts	Distribute educational materials, provide ongoing consultation, conduct educational outreach visits
[36] Hoogland, A. I.; Lechner, S. C.; Gonzalez, B. D.; Small, B. J.; Tyson, D. M.; Asvat, Y.; Barata, A.; Gomez, M. F.; Rodriguez, Y.; Jim, H. S. L.; et al. Efficacy of a Spanish-Language Self-Administered Stress Management Training Intervention for Latinas Undergoing Chemotherapy. *Psycho-oncology* **2018**, *27*, 1305–1311, doi:10.1002/pon.4673.	*n* = 240	Country of birth: Cuba, United States of America, Mexico, Colombia, Puerto Rico	Patients with breast, ovarian or lung cancer; mental health patients	Randomized controlled trial	≥18 years old; female; self-identified as Hispanic or Latina; able to speak and read in Spanish; diagnosed with cancer; scheduled to start outpatient intravenous chemotherapy for reasons other than symptom palliation; not having received chemotherapy in the previous two months; free of observable visual, auditory, psychiatric, or neurological disorders that would interfere with participation; and able to provide written informed consent	In-person individual, digital-based handouts, paper-based handouts, and video and audio files	Use of advisory boards and workgroups, distribute educational materials, tailor strategies, facilitation, conduct educational meetings, audit and provide feedback, promote adaptability
[43] McCusker J, Jones JM, Li M, et al. CanDirect: Effectiveness of a Telephone-Supported Depression Self-Care Intervention for Cancer Survivors. J Clin Oncol. **2021**;39(10):1150–1161. doi:10.1200/JCO.20.01802	*n* = 245	Canadian	Patients in cancer remission; breast, hematologic and lymphatic, genitourinary, gynecologic, other	Randomized controlled trial	Eligible participants were age 18 years or older, had completed primary treatment (surgery, radiation, and/or chemotherapy) for any cancer (adjuvant therapies permitted), had been diagnosed 1–10 years previously, were in remission, and had a Patient Health Questionnaire-9 depression score of 8–19, indicating mild to moderate depressive symptoms.	Web-based, paper-based handouts, and telephone-based guidance	Distribute educational materials, facilitation
[39] Nahm ES, McQuaige M, Steacy K, Zhu S, Seong H. The Impact of a Digital Cancer Survivorship Patient Engagement Toolkit on Older Cancer Survivors’ Health Outcomes. Comput Inform Nurs. **2025**;43(1):e01199. Published 2025 Jan 1. doi:10.1097/CIN.0000000000001199	*n* = 60	Americans (US)	Cancer-free subjects; breast cancer, head and neck cancer and colorectal cancer survivors	Randomized controlled trial	65 years or older, had completed primary cancer treatment with curative intent within 12 months prior to enrollment, Montreal Cognitive Assessment score of 26 or higher, had access to and the ability to use the Internet/email	Web-based, and digital-based communication	Distribute educational materials, facilitation Non-ERIC: reminder for participants
[31] Bennett MI, Mulvey MR, Campling N, et al. Self-management toolkit and delivery strategy for end-of-life pain: the mixed-methods feasibility study. Health Technol Assess. **2017**;21(76):1–292. doi:10.3310/hta21760	Phase 1 *n* = 19, Phase 2 *n* = 38, Phase 3 *n* = 19	United Kingdom	Patients with cancer and caregivers of patients with cancer; breast, lung, bowel, gynecological, pancreas, liver, bone, head/neck, metastatic sarcoma, or unknown	Randomized controlled trial	Patients in Phase 3 if they were: age ≥ 18 years, had been prescribed strong opioid analgesia, were living at home, were being cared for by specialist community palliative care services, were considered by the clinical team likely to survive beyond 6 weeks of follow-up, had the capacity to provide informed consent. Carers were eligible to take part in an end-of-study interview if they were the primary carers of a patient meeting the above inclusion criteria, and the patient whom they cared for had consented to their involvement.	In-person individual, paper-based handouts, and video and audio files	Conduct educational meetings, tailor strategies, distribute educational materials, provide ongoing consultation, assess for readiness and identify barriers and facilitators, promote adaptability, facilitation, develop and organize quality monitoring systems
[22] Lambert J, Loades M, Marshall N, et al. Investigating the Efficacy of the Web-Based Common Elements Toolbox (COMET) Single-Session Interventions in Improving UK University Student Well-Being: Randomized Controlled Trial. J Med Internet Res. **2025**;27:e58164. doi:10.2196/58164	*n* = 407	United Kingdom	Mental health patients	Randomized controlled trial	Currently registered undergraduate and postgraduate students in UK universities with internet access	Web-based	Distribute educational materials
[30] Yaffe MJ, McCusker J, Lambert SD, et al. Self-care interventions to assist family physicians with mental health care of older patients during the COVID-19 pandemic: Feasibility, acceptability, and outcomes of a pilot randomized controlled trial. PLOS One. **2024**;19(2):e0297937. doi:10.1371/journal.pone.0297937	*n* = 90	Canadian (64.4%)	Mental health patients	Randomized controlled trial	Age 65 or older, English or French speaking, and dwelling autonomously at home. Asymptomatic individuals who were interested in the intervention and were otherwise eligible were included in the study sample.	Paper-based handouts, digital-based handouts and telephone-based guidance	Distribute educational materials, facilitation, conduct educational meetings, develop and implement tools for quality monitoring, audit and provide feedback

## Data Availability

The complete research search strategy is available in the Appendix A. The study protocol can be accessed at OSF. The complete extraction table and data used for analyses can be found in the Supplementary Materials.

## Supplementary Materials

The following supporting information can be downloaded at: https://www.mdpi.com/article/10.3390/healthcare14142222/s1.

## Appendix A

### Search Procedures

This systematic review was conducted in accordance with the PRISMA guidelines and using the Covidence platform from 14 November 2023 to 12 December 2023. All articles published up to 2023 were included in this systematic review. A secondary search was conducted between 31 August and 4 September 2025 to review and capture articles published from 2024 to 2025. This review constituted a search of randomized control trials and quasi-experimental single-arm pre–post intervention trials using toolkits/toolboxes specifically addressing adults’ mental health and cancer patients and survivors.

The research team first used the following databases: PubMed, EBSCOhost, and Scielo.

The search strategy for PubMed includes “mesh”.

(((“Hispanic or Latino”[Mesh]) AND (“mental health”[Mesh])) AND (toolkit[Title/Abstract])) OR (Toolbox[Title/Abstract])
(((“Hispanic or Latino”[Mesh]) AND (“Cancer Survivors”[Mesh])) AND (toolkit[Title/Abstract])) OR (toolbox[Title/Abstract])
(((“Hispanic or Latino”[Mesh]) AND (“cancer patients”)) AND (toolkit[Title/Abstract])) OR (toolbox[Title/Abstract])
((((“Hispanic or Latino”[Mesh]) AND (“Oncologic Care”)) OR (Cancer care)) AND (toolbox[Title/Abstract])) OR (toolkit[Title/Abstract])
((((“Hispanic or Latino”[Mesh]) AND (“Delivery of Health Care”[Mesh])) OR (Cancer care)) AND (toolbox[Title/Abstract])) OR (toolkit[Title/Abstract])

The following keyword search with Boolean operators was used additionally in the databases on PubMed, and were used to search on EBSCOhost and Scielo:

((toolbox) OR (toolkit)) AND (mental health), (((toolbox) OR (toolkit)) AND (mental health)) AND (hispanic), (((toolbox) OR (toolkit)) AND (mental health)) AND (latino), (((toolbox) OR (toolkit)) AND (mental health)) AND (latinx), ((prevention toolbox) OR (prevention toolkit)) AND (mental health), ((toolbox) OR (toolkit)) AND (mental health prevention)
((toolbox) OR (toolkit)) AND (cancer), (((toolbox) OR (toolkit)) AND (cancer)) AND (hispanic), (((toolbox) OR (toolkit)) AND (cancer)) AND (latinx)
toolbox OR toolkit AND mental health AND cancer AND implementation, toolbox OR toolkit AND mental health AND cancer AND implementation strategies, toolbox OR toolkit AND mental health AND cancer AND implementation method, toolbox OR toolkit AND mental health AND cancer AND implementation resources, toolbox OR toolkit AND mental health AND cancer survivors AND implementation, toolbox OR toolkit AND mental health AND cancer survivors AND implementation strategies, toolbox OR toolkit AND mental health AND cancer survivors AND implementation method, toolbox OR toolkit AND mental health AND cancer survivors AND implementation resources, toolbox OR toolkit AND mental health AND cancer AND delivery, toolbox OR toolkit AND mental health AND cancer AND delivery method, toolbox OR toolkit AND mental health AND cancer AND delivery strategy, toolbox OR toolkit AND mental health AND cancer survivorship care, cancer patient toolbox AND caregiver toolbox AND implementation, caregiving AND cancer survivor AND mental health and toolkit, cancer survivor AND caregiver toolkit AND implementation, (((cancer survivor) AND (caregiver toolbox)) AND implementation, (((cancer patient) AND (caregiver toolbox)) AND (implementation)), (((cancer patient) AND (caregiver toolbox)) AND (implementation)) AND (mental health), (((cancer toolbox) AND (caregiver toolbox)) AND (mental health)) AND (implementation), ((((caregiver toolbox) AND (cancer)) AND (mental health))) AND (Implementation), ((caregiver toolkit) AND (mental health)) AND (implementation), ((cancer survivor caregiver toolkit) AND (mental health)) AND (implementation), ((cancer survivor toolkit) AND (implementation)), ((cancer toolkit) AND (implementation)), ((mental health toolkit) AND (implementation)), ((mental health toolkit) AND (implementation)) AND (cancer), (mental health toolbox) AND (implementation), (((implementation))) AND (mental health toolbox)

The research team also used the Google Scholar and Redalyc databases. The following keywords were used in the systematic review in the databases of Redalyc: toolbox OR toolkit AND mental health, Hispanic, Latinos (Latinas, Latinx), cancer patients, cancer survivors, cancer caregivers, mental health, NOT children. The following keywords will be used in the systematic review in the Google Scholar database: [+toolkit +toolbox +caregiver +mental health +cancer -children -child -prison -genetic -gene -meta-analysis -systematic review -nursing]
